# Supplementary material for: Green Bees: Reverse Genetic Analysis of Deformed Wing Virus Transmission, Replication, and Tropism
Source: Viruses. 2020 May 12;12(5):532. doi: 10.3390/v12050532 (PMC7291132; doi:10.3390/v12050532)
Supplement: Supplementary file 1 [file viruses-12-00532-s001.zip › Figure S1.pdf]

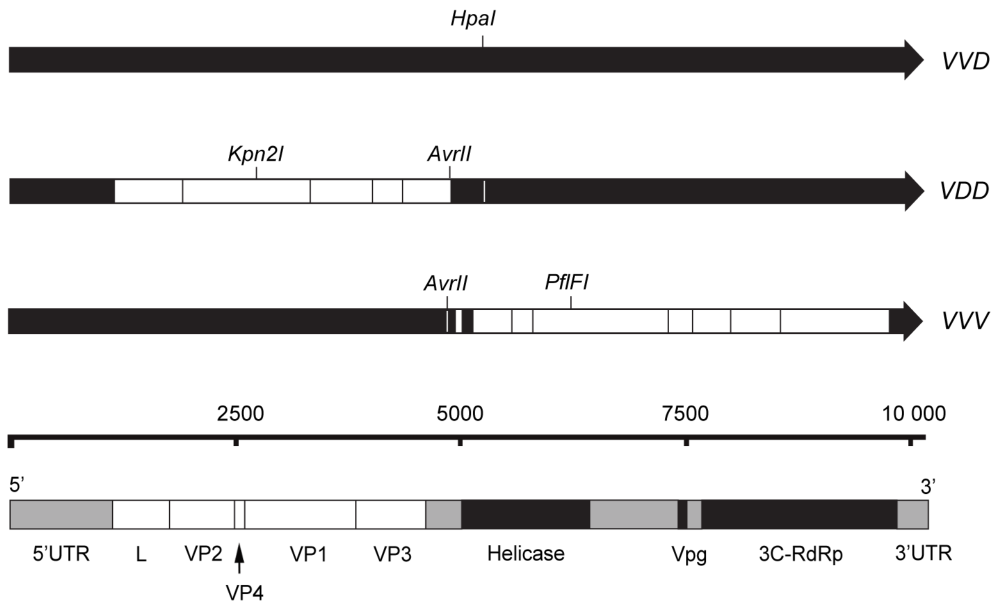

Figure S1. The DWV genome and cDNAs used in this study. Homologous regions between recombinant VVD sequence (VDV-1 VVD9) used as a backbone and derivative VDD (DWV A) and VVV (DWV B) variants are shown in black. Unique synonymous restriction sites introduced into each cDNA are indicated. Genomic RNA organization and encoded proteins are shown below.
