# Supplementary material for: Green Bees: Reverse Genetic Analysis of Deformed Wing Virus Transmission, Replication, and Tropism
Source: Viruses. 2020 May 12;12(5):532. doi: 10.3390/v12050532 (PMC7291132; doi:10.3390/v12050532)
Supplement: Supplementary file 1 [file viruses-12-00532-s001.zip › Figure S2.pdf]

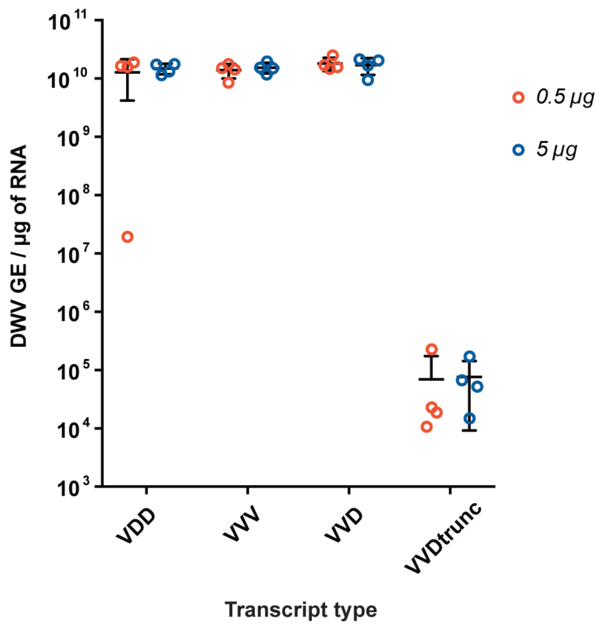

Figure S2. DWV accumulation in honey bee pupae injected with RG RNA transcripts. RT-qPCR analysis of honey bee pupae injected at white-eyed stage with 0.5 or 5 µg of RG DWV RNA for DWV A (VDD), DWV B (VVV) and recombinant (VVD) variants, VVDtrunc – control pupae injected with *in vitro* transcribed RNA prepared from a VVD cDNA template prematurely truncated within the RdRp coding region (nt 9233). Pupae were analyzed 72 h post-injection, data shown as individual values for each sample with error bars representing mean ±SD.
