# Supplementary material for: Green Bees: Reverse Genetic Analysis of Deformed Wing Virus Transmission, Replication, and Tropism
Source: Viruses. 2020 May 12;12(5):532. doi: 10.3390/v12050532 (PMC7291132; doi:10.3390/v12050532)
Supplement: Supplementary file 1 [file viruses-12-00532-s001.zip › Figure S3.pdf]

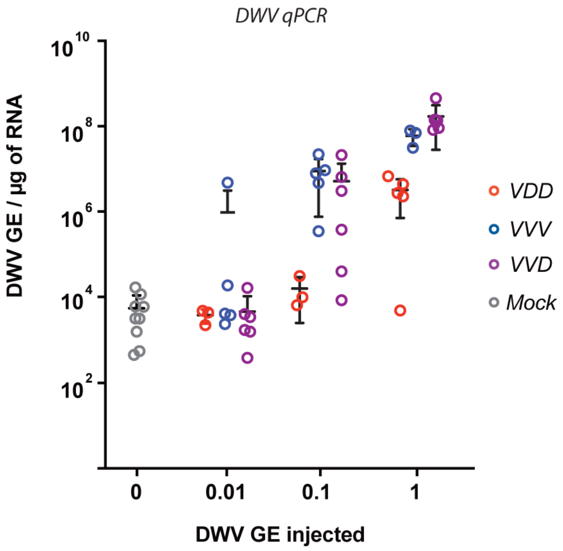

Figure S3. Inoculation of honey bee pupae with highly diluted RG-DWV. RT-qPCR analysis of DWV level in honey bee pupae (injected at white-eyed stage) 24 h post-injection with different amounts of DWV variants, "Mock" - not injected pupae. Each value corresponds to an individual sample analyzed, error bars show mean ±SD.
