# Supplementary material for: Green Bees: Reverse Genetic Analysis of Deformed Wing Virus Transmission, Replication, and Tropism
Source: Viruses. 2020 May 12;12(5):532. doi: 10.3390/v12050532 (PMC7291132; doi:10.3390/v12050532)
Supplement: Supplementary file 1 [file viruses-12-00532-s001.zip › Figure S4.pdf]

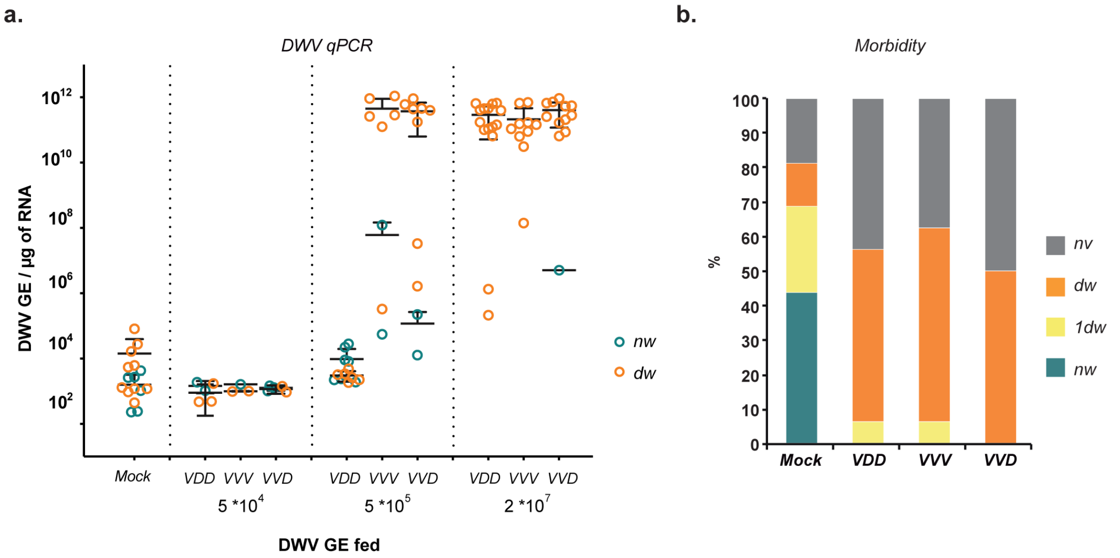

Figure S4. Morbidity of DWV variants in honey bees developed from infected larvae. (**a**) RT-qPCR analysis of DWV levels in newly emerged honey bees developed from honey bee larvae fed with plain diet (Mock) or infected with different amounts DWV variants and displaying normal ("nw") or deformed wing ("dw") phenotypes. Individual values for each sample are shown with dots, lines and error bars representing mean ±SD. (**b**) Percentage of visually normal ("*nw*"), partially deformed ("1dw"), deformed ("*dw*") and non-viable ("*nv*") honey bees developed from larvae fed diet containing $2*10^7$ GE of DWV (n=16 for each group).
