# Supplementary material for: Green Bees: Reverse Genetic Analysis of Deformed Wing Virus Transmission, Replication, and Tropism
Source: Viruses. 2020 May 12;12(5):532. doi: 10.3390/v12050532 (PMC7291132; doi:10.3390/v12050532)
Supplement: Supplementary file 1 [file viruses-12-00532-s001.zip › Figure S5.pdf]

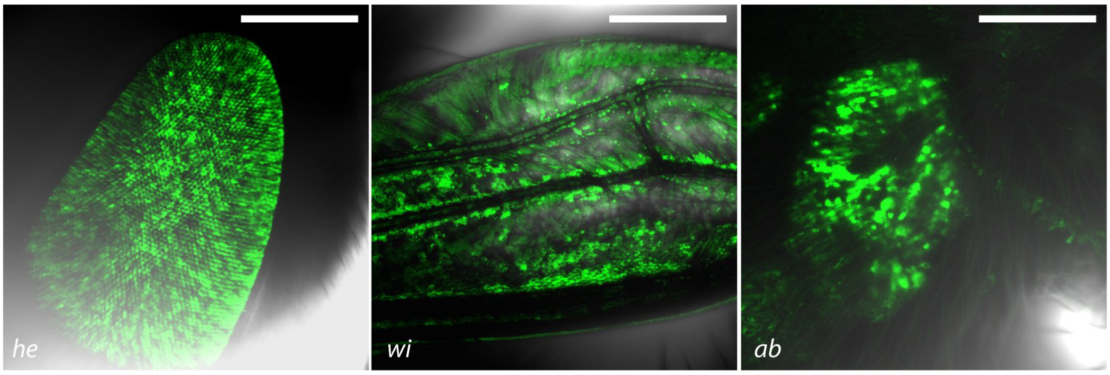

Figure S5. EGFP expression in honey bee infected with DWVE. Confocal microscopy imaging of DWV-produced EGFP in head (he), wings (wi), and abdomen (ab) of newly emerged live honey bee infected at pupa stage 7 days prior imaging. Panels show a combined image of the fluorescent and white field signals; scale bars correspond to 500 μm.
