# Supplementary material for: Green Bees: Reverse Genetic Analysis of Deformed Wing Virus Transmission, Replication, and Tropism
Source: Viruses. 2020 May 12;12(5):532. doi: 10.3390/v12050532 (PMC7291132; doi:10.3390/v12050532)
Supplement: Supplementary file 1 [file viruses-12-00532-s001.zip › Figure S6.pdf]

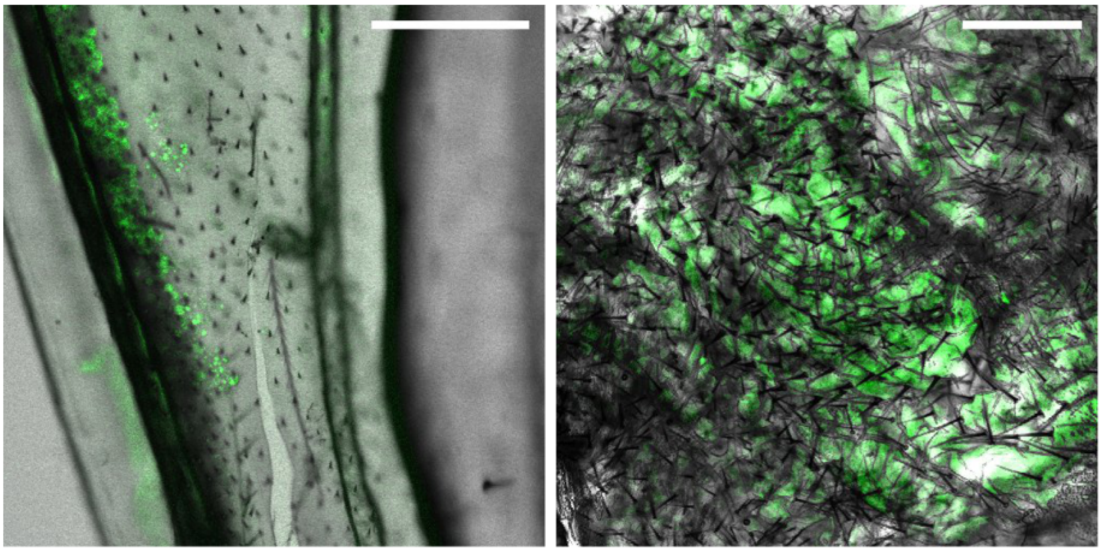

Figure S6. DWV localisation in honey bee wings. Confocal microscopy imaging of DWV-produced EGFP in wings of newly emerged honey bees infected at the pupal stage. Normal (left) and deformed (right) wings are shown as a combined image of the fluorescent and white field signals; scale bars correspond to 200 μm.
