# Supplementary material for: Green Bees: Reverse Genetic Analysis of Deformed Wing Virus Transmission, Replication, and Tropism
Source: Viruses. 2020 May 12;12(5):532. doi: 10.3390/v12050532 (PMC7291132; doi:10.3390/v12050532)
Supplement: Supplementary file 1 [file viruses-12-00532-s001.zip › Figure S7.pdf]

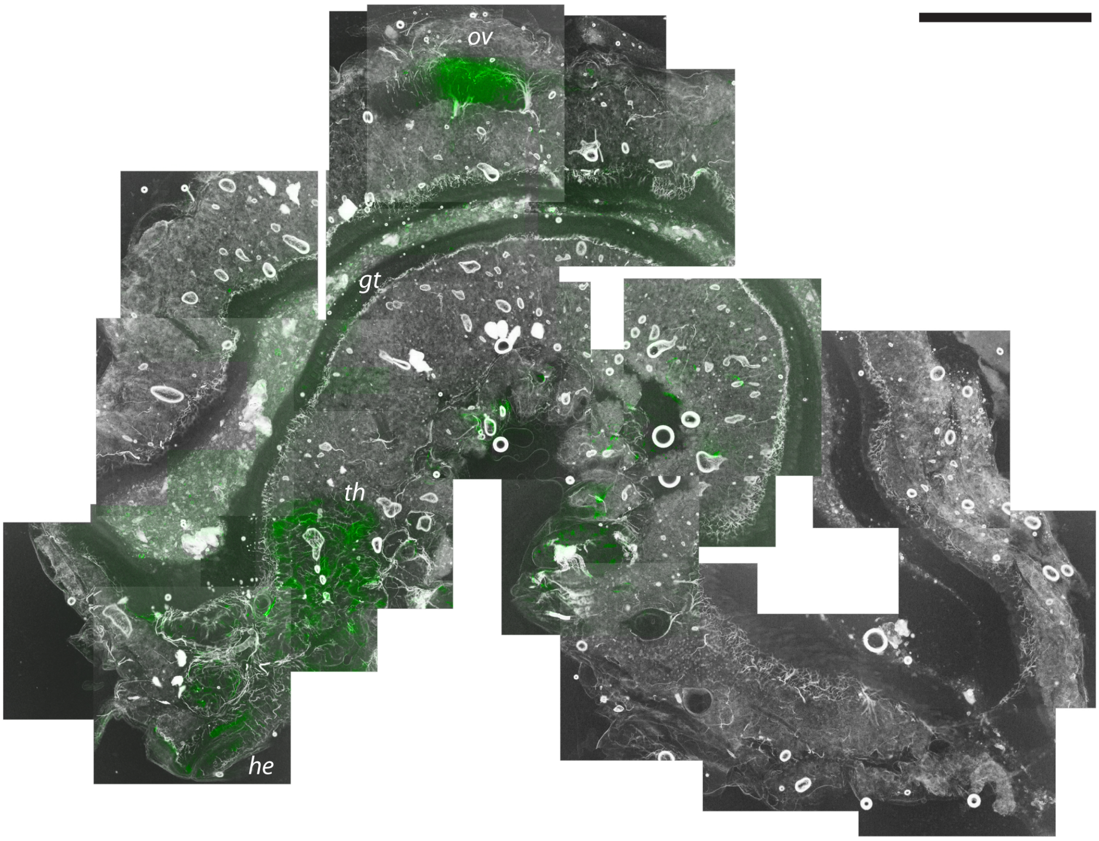

Figure S7. EGFP signal localisation in honey bee larva infected with DWV$_E$. Combined image obtained by confocal microscopy analysis of a single cryosection of the infected larva. Larva was sampled 6 days after feeding with 5*10^7 GE of DWV$_E$ inoculate; head - “he”, thoracic segments - “th”, rudimental ovary - “ov”, midgut - “gt”; each plane represents a composite of fluorescent signal z-stack and white-field image (inverted), scale bar shows 1 mm.
