# Supplementary material for: Green Bees: Reverse Genetic Analysis of Deformed Wing Virus Transmission, Replication, and Tropism
Source: Viruses. 2020 May 12;12(5):532. doi: 10.3390/v12050532 (PMC7291132; doi:10.3390/v12050532)
Supplement: Supplementary file 1 [file viruses-12-00532-s001.zip › Table S1.pdf]

Table S1. PCR primers used in this study.

Name	Sequence (5′-3′)	cDNA binding site	PCR product size	Reference
**DWV_RTPCR_F**	ATTGATCATTGTATGTTTACCTTCCCTTG	4021-4049		[32]
**DWV_RTPCR_R**	GCACGTAAGAGCTCGCTGCATA	5606-5627	1606 bp	[32]
**DWV_qPCR_F**	ATATCACTTGGCGACGCAAC	4950-4969		[32]
**DWV_qPCR_R**	CCAATCTTTAAATTGTTTCGGTTTTTGA GC	5098-5126	176 bp	[32]
**Kpn2I_F**	ATCCAGTGCAGGCAAAACCA	3288-3307	1545 bp	[32]
**Kpn2I_R**	TACCGCCCCGTACGTACACA	5247-5266		[32]
**PflFI_F**	TAATCCTGCAGCGCGACTCT	5247-5266	1530 bp	[32]
**PflFI_R**	GAACGCTCGTGGACATACGC	6757-6776		[32]
**VDD_RTPCR_F**	ACCATTAATTGCATCTGGATATAGA	4131-4155		*This study*
**VDD_RTPCR_R**	TAATGGATTAACAACACATTTTATGCCT	5649-5676	1545 bp	*This study*
**VVV_RTPCR_F**	GATAGCGTCAGGGTATCGG	4137-4155		*This study*
**VVV_RTPCR_R**	TTCACGACACACTTAATGCCC	5649-5669	1532 bp	*This study*
**DWV(-RNA)_RT**	CTTGGTTAGCTGTGTTGCAGTTGCTGTA GTTAAGCGGTTATTAGAA	4925-4947	120 bp	[10]
**DWV(-RNA)_RT R**	CTGTAGTTAAGCGGTTATTAGAA	4925-4947		[10]
**388 (adapter)**	CTTGGTTAGCTGTGTTGCAGTTG	-		[10]
***Apis mellifera* actin F**	AGGAATGGAAGCTTGCGGTA	919–938		[27]
***Apis mellifera* actin R**	AATTTTCATGGTGGATGGTGC	1099–1079	181 bp	[27]
**EGFP_qPCR_F**	GAGAAGCGCGATCACATGGT	639-659		*This study*
**VDD_VP2qPCR_R P**	AGATGTACTAGGATCTCGCTGAGTT	1860-1884	189 bp	*This study*
**VVV_VP2qPCR_RP**	TAATTCAACTTCACCTTCGCCATCTG	1808-1833	163 bp	*This study*
